# Should Doctors Know Their Patients’ Attachment Style? A Psychological Perspective and its Impact on Cardiac Surgery Outcomes

**DOI:** 10.21470/1678-9741-2019-0046

**Published:** 2020

**Authors:** Christiana Bithas, Amer Harky

**Affiliations:** 1 School of Medicine, University of Liverpool, Liverpool, UK.; 2 Department of Cardiothoracic Surgery, Liverpool Heart and Chest Hospital, Liverpool, UK.

**Keywords:** Depression. Stress, Psychological. Coronary Artery Bypass. Cardiac Surgical Procedures. Anxiety. Morbidity. Survivors

## Abstract

**Objective:**

To increase our understanding of the psychological attachment styles in order to develop a preventative strategy that could potentially improve patients’ perioperative outcomes.

**Methods:**

A comprehensive literature search was performed utilizing major electronic databases. The search was done from inception to January 2019. All of the relevant papers have been extracted and critically appraised in this review.

**Results:**

Understanding the psychological aspects of patients is crucial for a satisfactory postoperative outcome. Depression and anxiety have been shown to increase both mortality and morbidity after coronary artery bypass graft surgery, independently of medical factors, although the behavioural and biological mechanisms are poorly understood. Psychosocial assessment is an important part of the pre-transplant evaluation process. The majority of individuals undergoing a transplant have significant psychosocial problems and can either be deferred or denied the transplant until these psychosocial issues are approached and managed. Psychological distress has been shown to affect long-term prognosis of cardiac patients and as a result, it should be addressed during follow-up of cardiac arrest survivors due to cardiac cause. Several studies have considered different approaches and analyses of different psychological attachments, and the understanding of such parameters perioperatively could possibly minimise perioperatively complications.

**Conclusion:**

Since psychological distress affects long-term prognosis of cardiac surgery patients, it should be addressed during follow-up of cardiac arrest survivors due to cardiac cause.

**Table:** 

Abbreviations, acronyms & symbols	
CABG	= Coronary artery bypass grafting
CI	= Confidence interval
DSM-III-R	= Psychiatry Diagnostic and Statistical Manual of Mental Disorders-3^rd^ Edition Revised
HR	= Hazard ratio
IL-6	= Interleukin-6
OR	= Odds ratio
PTSD	= Post-traumatic stress disorder

## INTRODUCTION

Attachment theory draws on concepts from ethology, cybernetics, information processing, and developmental psychology and psychoanalysis in order to describe the way that children develop relationships through to adulthood^[1]^. Adult attachment styles can have an impact on cardiovascular disease and its relevant outcomes throughout the disease management process.

In the recent years, several studies established direct relationship and impact of preoperative psychological distress on the perioperative morbidities and mortalities of patients undergoing cardiac surgery^[2-4]^. Patients with anxious or avoidant attachment styles are at a greater risk than those with secure attachment styles^[5,6]^.

By increasing our understanding of the psychological attachment styles, we can begin developing a preventative strategy that could potentially improve patients’ perioperative outcomes.

### Classifications of Attachment Styles

In 1991, Ainsworth and Bowlby transformed the way we view the importance of parental relationships and their disruption through separation, deprivation, and bereavement^[7-11]^. Bowlby’s and Ainsworth’s studies in childhood development led to the conclusion that a secure strong attachment to their primary caregiver provided children with the foundations required to form a ‘securely-attached’ adult^[9,12]^. This so-called ‘secure attachment’ is critical to personal development, and failure to provide this in childhood results in a great deal of developmental energy to be expended by children in the search for stability and security. This can persist all the way to adulthood, resulting in non-secure attachment styles, as reported by Hazan and Shaver^[13-15]^.

The main differing attachment styles are outlined in Table 1, and we have adapted these to reflect on how they translate into clinical practice. It is important to acknowledge that individuals often fall within a range of the spectrum that can be of significant use during our daily work. These descriptors are based on the work of Bartholomew and Horowitz and on a review of studies by Pietromonaco and Barrett^[16,17]^.

**Table 1:** Attachment styles descriptors in adults.

Secure attachment	• It involves a positive view of self and others. • These individuals report greater satisfaction and adjustment in their relationships than people with other attachment styles. • Secure attachment results from caregivers who are emotionally available and respond appropriately to their children's emotional expressions and appropriately manage these expressions. • Medically, this can translate as individuals who take trust in their doctors, are compliant with the treatments offered, have healthy coping strategies when faced with medical adversities, and take more benefit from holistic approaches in patient care.
Anxious-preoccupied (insecure attachment)	• It involves having a negative sense of self and positive view of others. • It results from caregivers who failed to be emotionally available. • Medically, this translates as individuals who seek reassurance from their doctor but have the innate belief that they will not recover from illness. • Their anxiety is partly relieved through contact with their doctor but it does not resolve. • These are individuals who will present high levels of emotional expressiveness during consultations, emotional dysregulation, worry, and impulsiveness in decisions, which in turn affects their health.
Dismissive-avoidant (insecure attachment)	• These are individuals who have a positive view of self and a negative view of others. • They have adapted to become the primary caregivers from a young age, due to bereavement, failure of the primary caregiver to provide adequate care, or because they have experienced rejection during their development. • This desire of 'independence' results in an attempt to avoid attachment to another individual all together, for fear of experiencing this feeling of bereavement again. • Medically, these individuals tend to underreport medical symptoms, and may not seek medical attention at the initial stages of illness. • When these individuals do seek medical attention however, they require the need to feel in control of medical decisions and overly-exert their autonomy. • These individuals are more prone to post-traumatic stress disorder postoperatively.
Fearful-avoidant (insecure attachment)	• These are individuals who have a fluctuating view of themselves and others. • They are uncomfortable with the idea of getting close to others or having to depend on others. • These individuals deny their own emotions and have fluctuating levels of emotional expression. They do not trust the intentions of their attachments. • Medically, these individuals may present with a mixture of features ranging from those of anxious-preoccupied individuals to those of dismissive-avoidant.

Adult attachment styles were summarised by Fraley and Shaver^[18]^ as outlined in Table 2. They proposed that infant-caregiver relationships and adult relationships are based on the same framework, therefore leading adult attachments to imitate the forms of attachment formed during childhood. They expressed how the individual differences in adult attachment behaviour are reflections of the expectations and beliefs that people formed regarding their sense of self based on their childhood and close relationships.

**Table 2:** Implications of the adult attachment theory.

• Infant-caregiver relationships and adult relationships and their dynamics rely on the same biological system.
• The individual differences seen in adult relationships are similar to those seen in infant-caregiver relationships.
• The differences observed in adult attachment behaviour reflect the beliefs and expectations that individuals have formed about themselves and their close relationships on the basis of their previous attachments.
• The working models by which we form attachments are relatively stable and therefore can be assumed to reflect early caregiving experiences.

As we are biologically driven to form attachments with others, it serves as a coping mechanism, allowing adults to overcome illness^[19]^. Insecure attachment styles often lead to psychological distress, which in turn has a detrimental effect on health. Evidence suggests that accommodating and addressing patients’ sources of psychological distress not only reduce preoperative anxiety by making patients feel well-informed, but also increase their sense of control over recovery, which manifests as a facilitated physical recovery and has even been shown to reduce the incidence of postoperative hypertension in cardiac surgery by 32.5%^[20]^.

Most cardiovascular diseases are preventable through controlling and treating predisposing risk factors^[21]^. Extensive efforts have been made to show the effect that emotional vulnerability has in the manifestation of medical disorders and in cardiovascular disease in particular^[22]^. As our understanding of attachment style has evolved, we have begun to understand how poor attachment underpins the basis and instigates already well-known risk factors associated with cardiovascular disease such as: stress, anxiety, depression, anger, poor social support, marital status, educational level, poor coping mechanisms, including smoking and excessive alcohol intake, as well as other areas of lifestyle^[22-24]^.

### Cardiac Surgery and Attachment Styles

Patients undergoing cardiac surgery have been reported to react to the operation as a realistic threat to life, and it can be seen as a life-changing event. Presence of anxiety may lead to coping through denial as a maladaptive mechanism to overcome this threat^[24]^ Anxiously-attached individuals are more prone to experience this than other individuals. Depression is common in cardiac surgery patients, as physiological and behavioural factors such as endothelial dysfunction, platelet abnormalities, autonomic nervous system dysfunction, and reduced engagement in health-promoting activities link depression with adverse cardiac events^[25]^. It influences not only the recovery period but may also lead to increased morbidity and mortality rates^[26-30]^.

It is important to note that through knowledge of our attachment style, we are capable of dealing with the intense emotions, the pain of separation anxiety, and the grief that may have shaped our current worldview. It is possible to reappraise and reconstruct our inner representations of attachment relationships and shift our reaction to negative events^[31]^. For patients who may experience this sense of anxiety during pre- and postoperative periods, open conversation and psychological support may enable for this healing process to begin, and for the experience of surgery to be relabeled as a positive one^[32]^.

Apart from consequential depression and anxiety, patients may also develop cardiac psychosis. Dismissive-avoidant attachments are over-represented in psychosis with associations being found between a dismissive attachment pattern and positive psychotic symptoms, negative symptoms, and poor engagement with services; in individuals who do however seek help, impaired recovery from these negative symptoms has been found^[33]^. This is thought to be due to poor mentalization and deactivation of affect^[33]^.

Cardiac psychosis was described as early as 1806 by Corvisart as a triad of depression, restlessness, and irritability, which are accompanied by rising extremes of excitement alongside confusion, hallucinations, and delusions^[34]^. It is estimated that it has an incidence rate of up to 9%; this is potentially a preventable occurrence through preoperative psychiatric input, prompt diagnostic and therapeutic intervention, and implementing psychosocial means of support^[35,36]^.

The attachment style may not also predict psychiatric sequelae after cardiac surgery, but also contributes to the length of in-hospital stay and postoperative inflammatory response. A study by Kidd et al.^[5]^ examined the association between attachment and inflammatory responses in 167 patients undergoing coronary artery bypass grafting (CABG) surgery. Inflammatory markers measured through blood samples included interleukin-6 (IL-6), C-reactive protein, and tumour necrosis factor-alpha. The study demonstrated an association between increased circulating levels of IL-6 postoperatively in patients with anxious attachment styles only, supporting the hypothesis that attachment anxiety is important in physical recovery from surgery.

### Coronary Artery Bypass Grafting Surgery

CABG is one of the most scrutinized procedures in the literature relating psychology and cardiac surgery due to its association with neuropsychiatric sequelae^[37]^. Numerous studies have suggested that postoperative neuropsychological test performance is associated with cognitive deterioration five years post CABG^[38,39]^. In contrast, other studies attribute the cardiac risk factors as the contributors for the deterioration and suggest that this decline is modest^[40]^.

Despite this, it has been agreed upon that there is a cognitive deterioration after CABG surgery. The debate lies on whether this decline is due to psychological distress (depression, anxiety), and therefore it is potentially responsive to intervention, or whether it is due to the advancement of vascular pathology that potentially perpetuates the rate at which cerebral damage is sustained intraoperatively^[41]^.

Attachment styles determine the consequent reaction to a perceived or actual threat to the self, which can fall into three categories: personal threat (hunger, pain, death), environmental threat (challenging situation), and relational threat (separation from partner)^[42]^. This innate reaction has been studied over time by different authors and institutes internationally^[1,5,42,43]^. While traditionally it was thought that these were the main contributors, there is evidence that surgical procedures initiate the attachment response, and thus it can directly influence the outcomes.

Depression and anxiety have been shown to increase both mortality and morbidity after CABG surgery, independently of medical factors, although the behavioural and biological mechanisms are poorly understood^[43]^. Despite these factors not directly affecting neuropsychological functions, depression confers a risk for delirium occurrence^[43]^.

Unipolar depression is characterised by low mood and apathy, amongst other symptoms. The reported prevalence of unipolar depression among CABG surgery patients is thought to be between 15 to 20%^[43]^.

McKhann et al.^[44]^ reported occurrence of depression symptoms at 13% and 9% in a group of 124 CABG patients at postoperative one month and twelve months, respectively^[45]^. With patient satisfaction and outcome being so interlinked, these findings call for a preventative approach to mental health in patients undergoing cardiac surgery.

Furthermore, Peterson et al.^[45]^ explained how newly developed depressive symptoms result from the stressors of surgery that lead to a reactive-depression. In these cases, identifying depression is a complicated process as it may result in complicated somatic symptoms being experienced postoperatively.

Attachment anxiety has been shown to be a significant independent predictor of depression post CABG surgery in a study by Kidd et al.^[46]^. Their study showed that anxious-attachment style was a significant independent predictor of depressive symptoms at six to eight weeks following surgery (*P*=0.026). However, dismissive-avoidant style was not a significant predictor for the development of depressive symptoms (*P*>0.05). These results support the current literature evidence that suggests how levels of depression prevalence occur more frequently in individuals who are anxiously attached. It is, therefore, noted that there is strong association between anxious attachment, depression, and anxiety, which results from the negative working cognitive models that are activated during perceived times of threat, such as the perioperative period.

The recovery period for CABG patients can be difficult due to increased dependence on others, restricted mobility, and therefore overall poorer quality of life emotionally^[47]^. This can perpetuate a sense of hypervigilance in individuals with insecure attachment styles, particularly those who are anxiously attached. If this is prolonged, negative views of sense and self are perpetuated, and depression and anxiety may ensue.

Preoperative anxiety has also been associated with greater-all cause mortality (hazard ratio [HR]: 1.88; 95% confidence interval [CI]: 1.12, 3.17) and it was independent of age, renal disease, concomitant valve procedure, cerebrovascular disease, and peripheral vascular disease in the study by Tully et al.^[4]^. Taking these findings further, the study also showed that anxiety increases the odds for incident atrial fibrillation after CABG surgery (odds ratio [OR]: 1.09; 95% CI, 1.00 to 1.18; *P*=0.05). General anxiety disorder was also associated in a separate study by Tully et al.^[48]^ with acute in-hospital morbidity events such as renal failure, stroke, and myocardial infarction (OR: 3.26; 95% CI: 1.10, 9.67; *P*=0.03).

It is of interest to note that dismissive-avoidant attachment did not predict the development of anxiety or depression. In certain ways, this is not surprising, as individuals with dismissive-avoidant attachment styles are able to deactivate strategies of affect regulation, which in turn results in inhibition of appraisal and monitoring threat, supressed thoughts, and suppressed proximity seeking behaviours^[47]^. These individuals are as a result, by suppressing these means of seeking support, able to undergo acute stress episodes with low subjective levels of distress^[47]^.

It can be speculated that dismissive-avoidant attachment is momentarily protective on mood during stress situations such as CABG surgery. However, it is important to note that failure of expression of dismissive-avoidant attachment may be due to the accustoming of repressing emotions, which may eventually be expressed in the forms of anger, apathy, and guilt due to a sense of vulnerability; and in extreme cases, it may present as post-traumatic stress disorder (PTSD). The study by Dao et al.^[49]^ of 62,665 CABG patients showed that 9% of cases with existing PTSD and major depression diagnosis preoperatively had a greater risk of in-hospital mortality than patients with either PTSD or major depression alone (*P*=≤0.001). Other studies that simultaneously assessed both depression and anxiety symptoms showed that each negative emotional state foreshadowed a nearly two-fold increased risk of unplanned hospital readmissions^[50]^.

It is, therefore, of paramount importance that we begin an open discussion with our patients regarding their mental health and we screen for depression that can occur postoperatively and plan their management accordingly, in order to provide adequate and on-time recovery from such major stress procedure.

### Heart Valve Surgeries

Heart valve disease is becoming a public health matter due to increasing life expectancy and new surgical approaches being developed^[51]^. With heart valve disease progression, once symptomatic, it severely restricts the performance of daily living, leading to feeling fragile and poor performance, which in turn could lead to depression over time.

In a prospective study by Ho et al.^[52]^ of 648 patients undergoing either mitral or aortic valve replacements without CABG, 29.9% of the patients were found to be depressed at baseline. The unadjusted six-month mortality rate was 13.2% for depressed patients compared with 7.6% for non-depressed patients (*P*=0.03). In multivariable analyses, depression remained significantly associated with increased mortality (OR: 1.90; 95% CI: 1.07, 3.40; *P*=0.03). The findings in this study were consistent amongst patients undergoing aortic valve replacement, mitral valve replacement, and valve replacement without CABG.

In a separate prospective observational study by Faria et al.^[53]^ of 52 patients who were not known to have history of depression and underwent elective valve replacement, 52% of the patients had scores consistent with depression when assessed on the geriatric depression scale questionnaire postoperatively. Higher rate of postoperative complications, lower haemoglobin concentration, and longer in-hospital stay were found to be significant predictors for postoperative depression (*P*=0.045, *P*=0.015, and *P*=0.02, respectively). This study called for systematic screening and early prevention to be implemented in patients, considering the predictors to ensure better health outcomes.

A qualitative study by Kikkenborg Berg et al.^[51]^ of 10 patients undergoing heart valve replacement showed that patients reported overall a sense of suffering, weakness, and struggle to resume normality postoperatively.

The statements used by the patients to explain that despite quantifiable improvements in their medical condition, their mental perception did not allow them to see the benefit of the procedure were notable: “I improved my exercise capacity by 37% but I didn’t feel it”. Regarding bodily attention, patients complained of being overly aware of their body, working out to compensate for their scar or worrying about how their heart responded to activities. Despite these common themes, the study showed that over time patients gained vitality and returned to their daily life.

### Heart Transplantation

For most patients, the psychological stress and adjustments associated with heart transplantation originates prior to undergoing the procedure, at the discovery of the life-threatening illness. They undergo a process of grief, as described by Kubler-Ross, in an attempt to process the information, which includes: denial of the severity of illness, anger of fate, bargaining behavior, such as exercise in the hope of improving cardiac health, and depression when nothing stops the progression of the disease^[54]^.

In adults, changes in income, social circumstances, relationships, and sense of self may precipitate this grieving process until the patient comes to acceptance^[55]^.

Acceptance as a transplant candidate involves recognizing their own mortality. The wait for an appropriate donor is often described by patients as “interminable”. It has been found that the most overriding fear is that of dying before a donor heart is found; this in turn has a detrimental effect on everyday functioning, with some patients withdrawing from social relationships due to fear of leaving their home or areas they consider safe. Individuals with anxious attachment are more prone to this anxiety and depression, whilst individuals with dismissive-avoidant attachment are more vulnerable to developing PTSD.

Hospitalization after heart transplants also comes with its own psychological challenges. The period of psychological adjustment can be divided into three distinct phases characterized by initial euphoria, followed by depression, and finally achieving a sense of pseudo-acceptance, whereby patients are emotionally fragile but their self-confidence increases. These stages are explained in more detail in Table 3.

**Table 3:** Periods of psychological adjustment following a heart transplant.

Immediate postoperative period	• This is often described as a 'honeymoon period' or a time of euphoria where the threat of death is removed and symptoms such as angina disappear. • Acute delirium is often reported during this period.
Diagnosis and treatment of the first rejection episode	• This results in a period of depression. Patients may then develop a sense of hopelessness, guilt, or lack of control. • The onset of rejection has always been perceived by patients as hidden, and this notion can be augmented in patients receiving immunosuppression where the process of rejection has no clinical signs or symptoms and can be diagnosed only by myocardial biopsy. • Patients tend to master their medications and means of preventing infection; however, they may be less interested in, *e.g*., the biopsy technique, as they perceive it as a factor that they have no sense of control over.
The recovery or later postoperative period	• This begins with control or resolution of the first rejection episode. • This increases a sense of control and pseudo-acceptance in patients. • Self-confidence increases in this period however patients are more emotionally fragile if an adversity does occur.

Post-transplantation psychological adaptation is complex. In a study by Stukas Jr. et al.^[56]^ of 158 recipients and 142 family caregivers, a total of 10.5% of recipients met the Psychiatry Diagnostic and Statistical Manual of Mental Disorders-3rd Edition Revised (DSM-III-R) criteria for PTSD, and an additional 5% were probable cases. The DSM-III-R criteria are outlined in Table 4. Amongst the caregivers, 7.7% of patients met the full criteria and an additional 11% were probable cases. The study showed that being female, having a history of psychiatric illness, and having low levels of support increased this risk.

**Table 4:** Psychiatry Diagnostic and Statistical Manual of Mental Disorders-3rd Edition Revised criteria for post-traumatic stress disorder.

• Experience of a sufficiently stressful traumatic event.
• Re-experiencing memories of the event.
• Avoidance of trauma-associated stimuli and/or numbing of emotional response.
• Symptoms of increased arousal.
• Duration greater than one month.

Psychosocial assessment is an important part of the pre-transplant evaluation process. The majority of individuals undergoing a transplant have significant psychosocial problems and can either be deferred or denied the transplant until these psychosocial issues are approached and managed^[57]^. In a retrospective study by Schneekloth et al.^[57]^, a total of 164 heart transplant recipients were assessed to evaluate the outcomes of patients who initially had significant psychosocial problems but had addressed these and received a heart transplant. The study found out that after addressing the primary psychosocial issues before transplant, post-transplant length of stay, organ rejection, and survival rate were the same as of those without prior psychosocial concerns.

In a study by Dew et al.^[58]^, a cohort of 72 heart transplant recipients were followed longitudinally during their first year post transplantation. The study aimed to identify pre-transplant and perioperative psychosocial factors associated with increased vulnerability to and maintenance of psychological distress postoperatively. The study found out that anxiety and depression were elevated in the early post-transplant period, relative to normative data. One third of patients remained with high distress levels at follow-up assessments. Factors such as a personal history of psychiatric disorders, a younger age, lower social support from primary caregiver, poor self-esteem, exposure to major life events involving loss, a poor sense of mastery, and use of avoidance to cope with health problems were susceptible to these high levels of distress. This study supports that dismissive-avoidant attachment may contribute to distress postoperatively in heart transplant patients. In anxiously attached individuals, distress may manifest due to low social support, which may leave them with a sense of ‘abandonment’ similar to that experienced in childhood, perpetuating their anxiety.

In a study by Burker et al.^[59]^, a group of 50 heart transplant patients were evaluated to assess their coping mechanisms and how these related to their quality of life. The study found out that higher use of denial was associated with poorer mental health functioning. Denying the existence of a stressor is a common coping strategy of dismissive-avoidant individuals. This presented to be dangerous for post-transplant patients, due to their underreporting of symptoms, and this in turn led to a detriment in their quality of life.

Based on the abovementioned findings from several studies, addressing each patient’s psychological needs, we can aim to start reducing distress postoperatively in patients undergoing heart transplants.

### Circulatory Arrest

Survivors of cardiac arrest show high rates of mental illness, with more than 40% of them suffering from anxiety, 30% from depression, and 20% from PTSD^[60]^.

Anxiety is a normal response following a major cardiac event; patients with anxious attachments are more prone to develop anxiety after major cardiac events due to their negative affectivity. In its extreme form, PTSD can result in detrimental patient outcomes. Table 5 outlines the factors which act as predictors of PTSD following a major cardiac event.

**Table 5:** Factors predicting post-traumatic stress disorder after cardiac arrest.

• Cardiac-event factors including perception of threat to life, fear at the time of the event, severity of chest pain, illness comprehension, prior myocardial infarction, or cardiac hospitalisation.
• Psychiatric history such as depressive symptoms and prior hospitalisation.
• Lack of social support.
• Personality type: alexithymia, repressive coping style, and type D personality.
• Dissociation at the time of hospital admission and intensity of acute stress disorder.

It has been shown that cardiac arrest survivors are prone to develop re-experiencing and somatization, manifesting as flashbacks of the medical interventions and surgical procedures, distressing dreams, recalling the defibrillators’ shocks, and recalling the cardiac event to the point where it causes significant distress^[61]^. These patients are also prone to avoidance, and so avoiding any triggers that could ignite a memory of the event, the hospital, medication and situations in which they can experience their heart rate increasing, such as sexual activity or exercise. Patients with previous dismissive-avoidant attachment styles are more prone to this avoidance response due to their repressive coping style^[62]^.

It is estimated that the rates of this resulting PTSD vary across cardiac populations. Patients who have experienced a cardiac arrest have the highest rates of PTSD, followed by patients with implantable cardioverter defibrillators, and lastly patients who experience acute coronary syndrome^[63,64]^.

A meta-analysis of six prospective studies investigating the relationship between PTSD and cardiac disease, including patients free of cardiovascular disease at baseline, concluded that PTSD is independently associated with a higher risk for incident cardiovascular disease without (HR:1.55; 95% CI: 1.341, 79) and with adjustment for depression (HR:1.27; 95% CI: 1.08, -1.49)^[65]^.

Furthermore, there appears to be a dose-response relationship whereby individuals with higher levels of distress are at considerably greater risk of cardiotoxic effects^[66]^.

A prospective study of 1059 women demonstrated that those with more than five symptoms of PTSD were at 3.21 times the risk of incident coronary heart disease compared to those with no symptoms^[67]^; these results have been widely reproducible in other studies^[68,69]^.

In regard to other psychological and psychiatric sequelae, in a retrospective Dutch study of 63 survivors with a mean follow-up of three years after open heart surgery, 74% of patients reported low participation levels in society, over 50% of patients reported severe fatigue, 38% reported feelings of anxiety/depression, and 24% of patients reported a decreased quality of life^[70]^. A better outcome was reported in an Australian study of 106 survivors five years after cardiac arrest where 84% of patients were living at home independently, and only 11% reported marked or severe disability^[71]^. Despite this, 71% of patients reported varying degrees of anxiety.

Therefore, since psychological distress affects long-term prognosis of cardiac patients in general, it should be addressed during follow-up of cardiac arrest survivors due to cardiac cause.

## CONCLUSION

Understanding patients’ perception of recovery following open heart surgery is of paramount. Despite improvements in cardiac surgery techniques and practice, patients' reactions to these events appear to remain unchanged. Introducing a lifeworld perspective based on patients’ own experience, attachment style, and expressions can not only improve patients’ perception of recovery but also overall patients’ outcomes in short and mid-terms.

**Table:** 

Author's roles & responsibilities	
CB	Substantial contributions to the conception or design of the work; or the acquisition, analysis, or interpretation of data for the work; drafting the work or revising it critically for important intellectual content; agreement to be accountable for all aspects of the work in ensuring that questions related to the accuracy or integrity of any part of the work are appropriately investigated and resolved; final approval of the version to be published
AH	Substantial contributions to the conception or design of the work; or the acquisition, analysis, or interpretation of data for the work; drafting the work or revising it critically for important intellectual content; agreement to be accountable for all aspects of the work in ensuring that questions related to the accuracy or integrity of any part of the work are appropriately investigated and resolved; final approval of the version to be published
